# Large reversible magnetocaloric effect in antiferromagnetic Ho_2_O_3_ powders

**DOI:** 10.1038/s41598-017-14279-y

**Published:** 2017-10-24

**Authors:** A. Boutahar, R. Moubah, E. K. Hlil, H. Lassri, E. Lorenzo

**Affiliations:** 1 LabSIPE, Ecole Nationale des Sciences Appliquées, Université Chouaib Doukkali d’El Jadida, El Jadida, Plateau 24002 Morocco; 2 LPMMAT, Université Hassan II-Casablanca, Faculté des Sciences Ain Chock, BP 5366 Mâarif-Casablanca, Morocco; 3 Institut Néel, CNRS, Université Grenoble Alpes, 25 rue des Martyrs, BP 166 38042 Grenoble cedex 9, France

## Abstract

Giant magnetocaloric materials are highly promising for technological applications in magnetic refrigeration. Although giant magnetocaloric effects were discovered in first-order magnetic transition materials, it is accompanied by some non-desirable drawbacks, such as important hysteretic phenomena, irreversibility of the effect, or poor mechanical stability, which limits their use in applications. Here, we report the discovery of a giant magnetocaloric effect in commercialized Ho_2_O_3_ oxide at low temperature (around 2 K) without hysteresis losses. Ho_2_O_3_ is found to exhibit a second-order antiferromagnetic transition with a Néel temperature of 2 K. At an applied magnetic field change of 5 T and below 3.5 K, the maximum value of magnetic entropy change $(-\Delta {{\rm{S}}}_{M}^{max})$, the refrigerant capacity (RC) were found to be 31.9 J.K^−1^.kg^−1^ and 180 J.K^−1^, respectively.

## Introduction

Cryocoolers able of cooling at low temperature (1.8–20 K) are widely utilized in different applications. As examples, they are used in hydrogen and helium liquefactions, superconducting quantum interference device (SQUID), medical instrumentation and diverse scientific research technologies. Superconducting magnet materials producing strong magnetic fields are broadly utilized in medicine and laboratories for scientific aims. Generally, liquid helium is used for cooling them, due to the fact that superconducting magnets exhibit low superconducting order temperature transition. However, liquid helium is expensive and scarcer which is not convenient from the economic point of view. Consequently, low-energy consumption cryocoolers are required. Magnetic refrigeration based on magnetocaloric effect (MCE) is a promising solution for refrigeration at low temperature^1–7^. Recently, considerable efforts were devoted to rare-earth based intermetallic compounds for low temperature magnetic refrigeration and some of them exhibit good MCE properties^8–16^. One of the main challenges in developing magnetic cryocoolers is to find a suitable working temperature range, large reversible magnetocaloric effect with low magnetic hysteresis losses. To date, the common used materials for cryogenic refrigeration^17–19^ based on magnetocaloric effect^20–22^ are hydrated salts. Such materials are used in low temperature cooling systems for detectors in space mission or laboratory facilities. The performance of an adiabatic demagnetization refrigerator (ADR)^23^ is critically dependent on the design and construction of these salt pills that produce cooling. However, the only available salts refrigerants present some drawbacks because they are hydrated, which requires to be encapsulated in a hermetic container to prevent dehydration. Furthermore, hydrated salts are fabricated by growth which is not appropriate with industrial processes because it is very time consuming.

One can notice that for room temperature applications giant magnetocaloric effects (MCE) were reported in materials with a first-order magnetic transition (FOMT) such as LaFe_13−x_Si_x_^24–29^ Gd_5_(Si,Ge)_4_^6^ and others^30–32^. However, FOMTs occur in a narrow temperature window and are often accompanied with some non-desirable drawbacks such as the irreversibility of the MCE, very large thermal and magnetic-field hysteresis losses^33^ and their high material cost. We note that very recently, the irreversibility of the MCE has been overcome in FOMT FeRh thin films^34^ using dual-stimulus multicaloric cycle. It is known that magnetic materials with a second-order magnetic transition (SOMT) lack a very large (−ΔS_M_),^35–40^ but they do present some advantages such as low magnetic hysteresis and tunable order temperature by varying composition. In this work, we found that commercialized Ho_2_O_3_ powders presents a giant magnetocaloric effect without magnetic hysteresis losses at low temperature.

## Experimental Details

Holmium (II) oxide Ho_2_O_3_ polycrystalline powders (≥99.9% purity) was provided by Sigma Aldrich factory and heated at 1200 K for 48 hours. The crystalline structure was checked by x-ray diffraction (XRD) using D5000 Siemens diffractometer with Co-K_α1_ radiation (λ = 1.5406 Å). Vibrating sample magnetometer (VSM)-quantum design was used to perform magnetic measurements at temperature ranging from 1.8 to 50 K, under an external applied magnetic field up to 5 T.

## Results and Discussion

**Figure 1:**
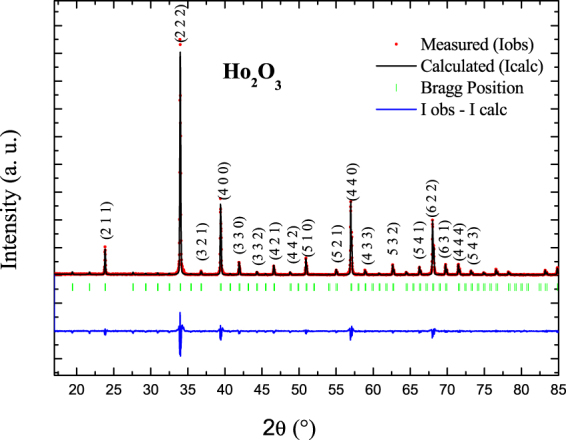
Rietveld refined powder XRD patterns of the Ho_2_O_3_ compound.

**Figure 2:**
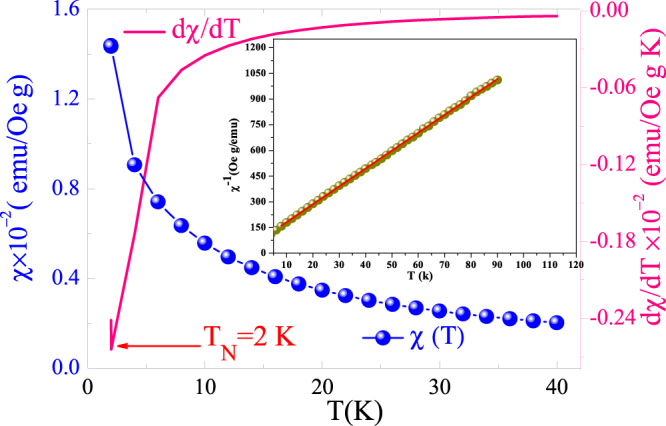
Change of the magnetic susceptibility and dχ/dT as a function of temperature recorded at an applied magnetic field of 0.05 T for Ho_2_O_3_ compound. Inset shows the inverse susceptibility versus temperature, symbol and solid line represent the experimental and linear fit obtained using Curie Weiss law.

The XRD pattern of Ho_2_O_3_ powders is displayed in Fig. 1. Different diffraction peaks can be observed, indicating a polycrystalline character of the sample. All the diffraction peaks can be indexed according to the bixbyite structure, which is in agreement with the data found in the literature^41^. The lattice parameters were determined using Rietveld refinement and were found to be *a* = *b* = c = 10.6186 Å with R_WP_ = 10.1%, R_P_ = 12.18% and **χ** ^2^ = 4.82. The absence of any additional peak in the XRD pattern demonstrates that there are no spurious phases in the detection limit of XRD experiments. Figure 2 displays the temperature dependence of the magnetic susceptibility recorded at an applied magnetic field of 0.05 T for the Ho_2_O_3_ compound. As can be observed, the sample shows a decrease in magnetic susceptibility with increasing temperature which is associated with the antiferromagnetic interaction in Ho_2_O_3_ in agreement with previous reports^41^. In order to determine the Néel temperature (T_N_), we display in the inset of Fig. 1(a) the $\chi =\frac{C}{T-{\theta }_{P}}.$ versus T plot. The T_N_ is defined as the inflection point of derivative and it is around 2 K. Inset of Fig. 2 shows the change of magnetic susceptibility (*χ* ^−1^) as a function of temperature. In the paramagnetic region, *χ* ^−1^(T) was fitted using the classical Curie-Weiss law:

1 $$\chi =\frac{C}{T-{\theta }_{P}}.$$

where C is the Curie constant and *θ*_*p*_ is the Curie-Weiss temperature.

From the linear fit, the C and *θ*_*p*_ parameters were obtained. The negative (*θ*_*p*_ = −7 K) value confirms the presence of an antiferromagnetic interaction. The C constant is related to the effective paramagnetic moment by the following relation $C=\frac{{N}_{A}{\mu }_{{eff}^{2}}}{3{K}_{B}}$; where N_A_ = 6.023 10^23^ mol^−1^ is the Avogadro number, µ_B_ = 9.274 10^−24^ (A/m^2^) is the Bohr magneton and k_B_ is the Boltzmann constant. From the determined C parameter, we have deduced the real effective moment of Ho value which was found to be ${\mu }_{eff}^{exp}$ = 11.8 µ_B_. The experimental effective paramagnetic moment ${\mu }_{eff}^{exp}$ is higher than the theoretical value (${\mu }_{eff}^{the}$ = 10.6 µ_B_), which could be attributed to the crystal field effects which favors a high spin configuration^41^.

**Figure 3:**
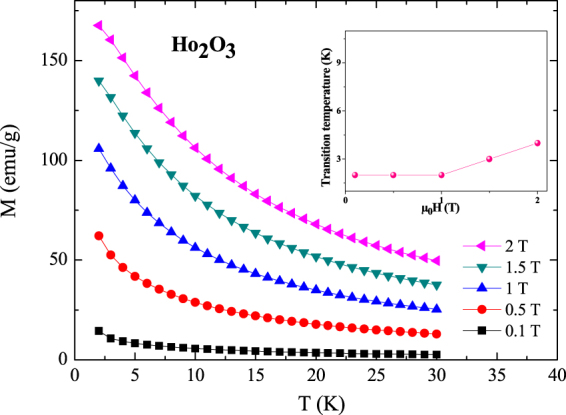
Temperature dependence of magnetization obtained at different magnetic fields (μ_0_H) up to 2 T of the Ho_2_O_3_ compound. Inset shows the magnetic field dependence of transition temperature.

Figure 3 shows the temperature dependence of the magnetization at different applied magnetic fields. We display in the inset of Fig. 3 the magnetic field dependence of transition temperature. As shown, the magnetic transition is sensitive to the high magnetic field. Sharp change of the M(T) curve can be observed with increasing temperature at low magnetic field, while the increase of the applied field leads to a broader distribution of the M (T) curve. With increasing applied magnetic field more magnetic moments are forced to follow the direction of the applied field, which induced a broader distribution of the M(T) curve.

**Figure 4:**
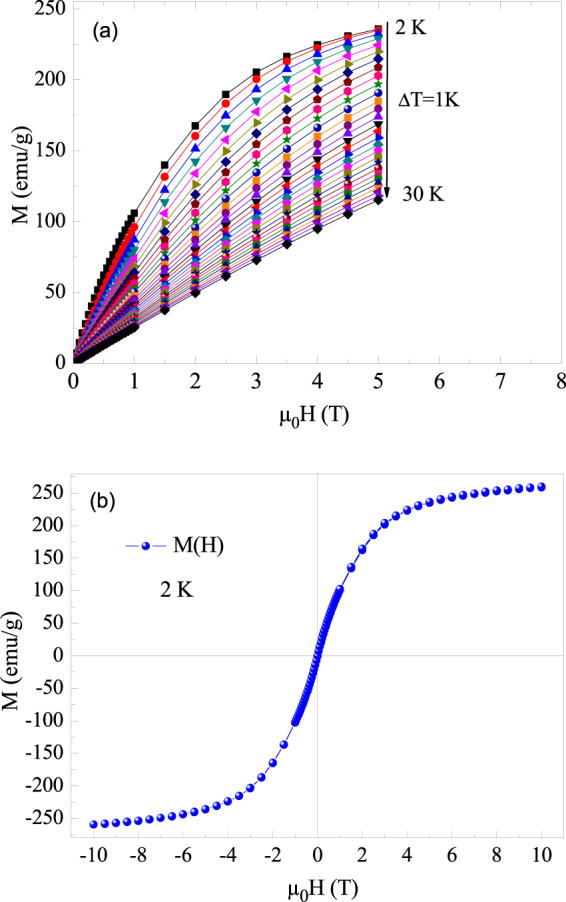
(**a**) Isothermal magnetization curves obtained at different temperatures from 2 to 30 K with an increment of 1 K of the Ho_2_O_3_ compound. (**b**) The full magnetization curve measured at transition temperature.

**Figure 5:**
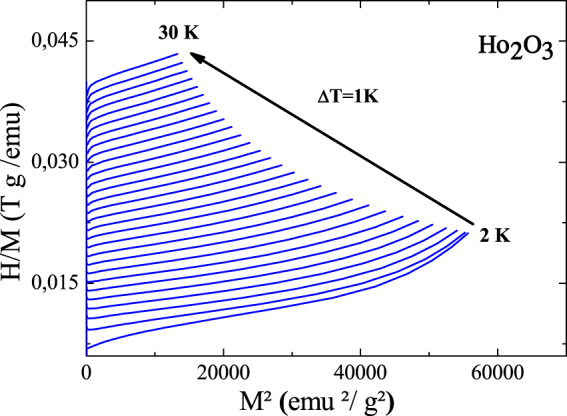
Arrott plots of the Ho_2_O_3_ compound.

Isothermal magnetization curves were measured at various temperatures (Fig. 4). The gradual evolution of these curves to linear behavior characterizes an increase of the paramagnetic contribution above T_N_. Figure 4(b) presents the magnetic hysteresis loop of the Ho_2_O_3_ powder recorded at 2 K. The hysteresis loop is closed and completely reversible. These properties are highly suitable for magnetic refrigeration^42^. In order to investigate the nature of the magnetic phase transition, Arrott plots (H/M versus M^2^) were studied (Fig. 5). According to the Banerjee criterion^43^, a magnetic transition is the first-order when the slope of Arrott curves is negative, whereas it will be second-order when the slope is positive. As can be observed, positive slopes are observed for all temperatures which show that the H_2_O_3_ compound undergoes a SOMT.

The magnetocaloric effect can be related to the magnetic properties of the material through the thermodynamics Maxwell’s relation. It has been calculated in terms of isothermal magnetic entropy change using isothermal magnetization obtained at various temperatures (Fig. 5). According to the thermodynamically theory^6^, the isothermal magnetic entropy changes associated with a magnetic field change is given by:

2 $${\rm{\Delta }}{S}_{M}(T,{\rm{\Delta }}H)={S}_{M}(T,H)-{S}_{M}(T,0)={{\int }_{0}^{{\mu }_{0}{H}_{MAX}}(\frac{\partial S(T,H)}{\partial H})}_{T}H$$

From the Maxwell’s thermodynamic relation

3 $${(\frac{\partial S(H,T)}{\partial H})}_{T}={(\frac{\partial M(H,T)}{\partial T})}_{H}$$

One can obtain the following expression

4 $${\rm{\Delta }}{S}_{M}{(T,{\rm{\Delta }}H)}_{{\rm{\Delta }}H}={{\int }_{0}^{{\mu }_{0}{H}_{max}}(\frac{\partial M(H,T)}{\partial T})}_{H}dH.$$

Where µ_0_H_max_ is the maximum external field.

**Figure 6:**
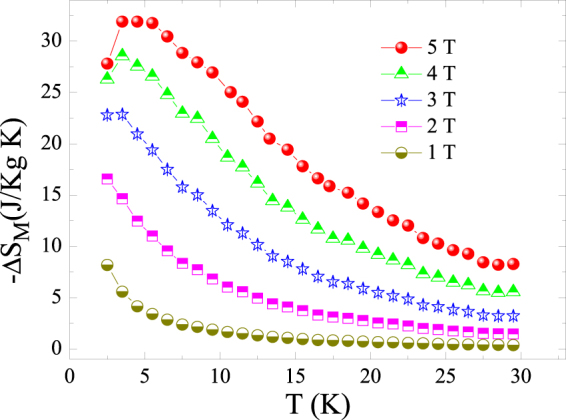
Temperature dependence of magnetic entropy change (−ΔS_M_) under different magnetic field changes 1, 2, 3, 4, and 5 T of the Ho_2_O_3_ compound.

Figure 6 displays the temperature dependence of the magnetic entropy change of Ho_2_O_3_ powders obtained at different applied magnetic field changes (1, 2, 3, 4, and 5 T). For all fields, the (−ΔS_M_) curves show a maximum at around 3 K, which it is close to T_N_. We note that for a second-order phase transition the (−ΔS_M_)(T) should show a peak with a maximum around T_N_, however, since the T_N_ of the sample is too low (2 K), we only observe half peak of (−ΔS_M_)(T). The peak magnitude increases when ΔH increases, from 8.2 to 31.9 J/kgK with increasing applied magnetic field change from 1 to 5 T, respectively. The large magnetocaloric effect in H_2_O_3_ can be understood by its high magnetization associated with its sharp magnetization change at the antiferromagnetic-paramagnetic and the presence of crystal field effects above the transition.

Other important parameters of refrigerant materials are the refrigerant capacity RC and the adiabatic temperature change ΔT_ad_. According to Wood and Potter^44^ the RC of a reversible refrigeration cycle operating between T_h_ and T_c_ (temperatures of the hot and cold reservoirs, respectively) is defined as RC = (**−** *Δ* ***S***_***M***_) × ΔT, where (**−** *Δ* ***S***_***M***_), is the magnetic entropy change at the hot and cold ends of the cycle and ΔT = T_h_ − T_c_. For magnetic field changes of 0–4 T and 0–5 T, the values of RC were estimated to be 165 and 180 J/kg, respectively.

ΔT_ad_ can be calculated from magnetization and heat capacity measurements C_p_^6^

5 $$\Delta {T}_{ad}={\mu }_{{\bf{0}}}{\int }_{{\bf{0}}}^{{\mu }_{{\bf{0}}}H}\frac{T}{{C}_{p}}{(\frac{\partial M}{\partial T})}_{H}dH$$

**Figure 7:**
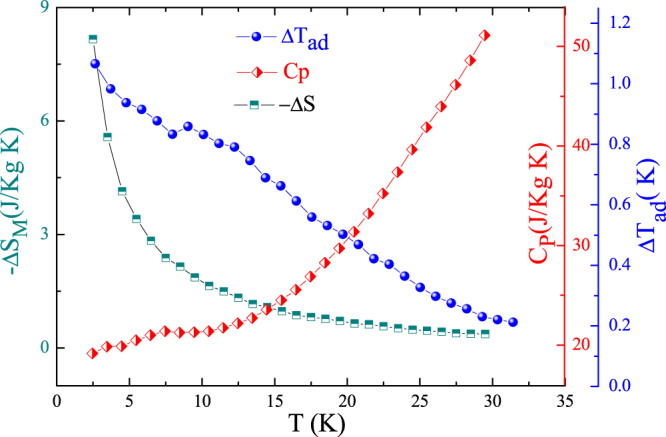
Temperature dependence of magnetic entropy change (−ΔS_M_), heat capacity and adiabatic temperature change ΔT_ad_ under magnetic field changes 1 T of the Ho_2_O_3_ compound.

The temperature dependence of ΔT_ad_ and heat capacity for magnetic field changes of 1 T is shown in Fig. 7. It can be seen that ΔT_ad_ increases with decreasing temperature. The maximum values of adiabatic temperature change $({\rm{\Delta }}{T}_{ad}^{max})$ reaches 1.08 K for a magnetic field change of 1 T.

In order to examine the usefulness of the Ho_2_O_3_ compound reported in this work, we made a comparative study of *T*_*N*,C_, $(-{{\rm{\Delta }}S}_{M}^{max})$, RC with other magnetocaloric materials found in literature with low temperature magnetic transitions. The comparison is summarized in Table 1. It can be concluded that $(-{{\rm{\Delta }}S}_{M}^{max})$ is larger or comparable to those of reported potential magnetic refrigerant materials,^13–15,45–51^. However, the ordering temperature of Ho_2_O_3_ compound is smaller than the other materials. We note that the RC factor is comparable or smaller than those of the materials reported in Table 1. The giant values of (−∆S_M_), the low-cost and fast way of preparation suggest that this compound is one suitable candidate as a magnetic refrigerant in low temperature range (around 2 K).

**Table 1:** Magnetic ordering temperature (T_N,C_), maximum values of (−ΔS_M_^max^) and refrigerant capacity (RC) under the magnetic field change of 5 T of the Ho_2_O_3_ compound and some potential magnetic refrigerant materials.

Material	***T***_C,N_ (K)	−ΔS_m_^max^ (J/kg K)	RC (J/kg)	Ref.
Ho_2_O_3_	2	31.9	180	This work
Ho_3_Al_2_	40	18.7	—	47
Ho_30_Y_26_Al_24_Co_20_	5.5	10.76	241	48
TmZn	8.4	26.9	214	49
ErRuSi				
	8	21.2	—	50
ErMn_2_Si_2_	4.5	25.2	—	51
HoCu_2_O_5_	14	9.2	—	13
HoNiAl_2_	7.5	14	171	14
TmZnAl	2.8	9.4	149	15

## Conclusion

In this paper, we have studied the magnetic and magnetocaloric properties of Ho_2_O_3_ powders. Magnetic measurements have shown the presence of an antiferromagnetic–paramagnetic transition around 2 K and a giant magnetic entropy change with second-order magnetic transition. Strong influence of crystal field effect is also observed in magnetic properties as well as magnetocaloric effect. Our study demonstrates that the Ho_2_O_3_ material could be considered as a potential candidate for magnetic refrigeration applications at low temperature (around 2 K).
